# Promoting Independence in Dementia (PRIDE): protocol for a feasibility randomised controlled trial

**DOI:** 10.1186/s13063-019-3838-x

**Published:** 2019-12-11

**Authors:** Aisha Shafayat, Emese Csipke, Lucy Bradshaw, Georgina Charlesworth, Florence Day, Phuong Leung, Esme Moniz-Cook, Alan A. Montgomery, Steve Morris, Gail Mountain, Reuben Ogollah, Kirsty Sprange, Lauren Yates, Martin Orrell

**Affiliations:** 1 Nottingham Clinical Trials Unit, University of Nottingham, Building 42, University Park, Nottingham, NG7 2RD UK; 2 Division of Psychiatry, University College London, 6th Floor, Maple House, 149 Tottenham Court Road, London, W1T 7NF UK; 3 North East London NHS Foundation Trust, Goodmayes Hospital, 1st Floor, Maggie Lillie Suite, Ilford, IG3 8XJ UK; 4 Psychology Ageing & Dementia Care Research, Faculty of Health Sciences, Department of Psychological Health, Wellbeing and Social Work, University of Hull, Hull, HU6 7RX UK; 5 Department of Applied Health Research, University College London, 1-19 Torrington Place, London, WC1E 7HB UK; 6 Faculty of Health Studies, University of Bradford, Richmond Road, Bradford, BD7 1DP UK; 7 Division of Psychiatry and Applied Psychology, University of Nottingham, Room D07, Institute of Mental Health Innovation Park, Triumph Road, Nottingham, NG7 2TU UK

**Keywords:** Dementia, Self-management, Feasibility trial

## Abstract

**Background:**

Memory services often see people with early stage dementia who are largely independent and able to participate in community activities but who run the risk of reducing activities and social networks. PRIDE is a self-management intervention designed to promote living well and enhance independence for people with mild dementia.

This study aims to examine the feasibility of conducting a definitive randomised trial comparing the clinical and cost-effectiveness of the PRIDE intervention offered in addition to usual care or with usual care alone.

**Methods/design:**

PRIDE is a parallel, two-arm, multicentre, feasibility, randomised controlled trial (RCT). Eligible participants aged 18 or over who have mild dementia (defined as a score of 0.5 or 1 on the Clinical Dementia Rating Scale) who can participate in the intervention and provide informed consent will be randomised (1:1) to treatment with the PRIDE intervention delivered in addition to usual care, or usual care only. Participants will be followed-up at 3 and 6 month’s post-randomisation. There will be an option for a supporter to join each participant. Each supporter will be provided with questionnaires at baseline and follow-ups at 3 to 6 months. Embedded qualitative research with both participants and supporters will explore their perspectives on the intervention investigating a range of themes including acceptability and barriers and facilitators to delivery and participation. The feasibility of conducting a full RCT associated with participant recruitment and follow-up of both conditions, intervention delivery including the recruitment, training, retention of PRIDE trained facilitators, clinical outcomes, intervention and resource use costs and the acceptability of the intervention and study related procedures will be examined.

**Discussion:**

This study will assess whether a definitive randomised trial comparing the clinical and cost-effectiveness of whether the PRIDE intervention offered in addition to usual care is feasible in comparison to usual care alone, and if so, will provide data to inform the design and conduct of a future trial.

**Trial registration:**

ISRCTN, ISRCTN11288961, registered on 23 October 2019, http://www.isrctn.com/ISRCTN12345678 Protocol V2.1 dated 19 June 2019.

## Background

Dementia challenges society, individuals and can have profound effects on family carers. People with cognitive impairment and dementia can experience ‘excess disability’ due to stigmatisation, loss of independence and a sense that relatives seek to take over their tasks and run their lives [1, 2]. Low expectations mean that staff and carers often don’t encourage people with dementia to use their skills or learn new things, further contributing to decline. Hobbies and interests are often lost early in the disease process [3]. A review indicated that provision of information and advice can improve quality of life in dementia [4], but this needs to be delivered with ease as alow-cost intervention to improve support and care.

Self-management interventions are a core part of current UK health policy and provision for long-term (chronic) conditions [5]. Such interventions can be delivered and received in a variety of ways (individually and in a group) and may be computer-assisted, mail-delivered, telephone-based or in a face-to-face format [6]. The aim is to live well with chronic illness, managing one’s condition and its emotional impact, and maintain as active a life as possible [7]. The limited research from self-management programmes for people with dementia [6, 8, 9] suggests that self-management programmes may address the current “care gap” in supporting people living with early stage dementia [10].

The PRIDE study comprises five interrelated strands investigating early/mild dementia. The first two strands involved an epidemiological investigation into the risk and protective factors associated with dementia in two large longitudinal databases of older adults in England and Brazil [11] and a qualitative exploration of social discourses of dementia focusing on the themes of memory and independence, involving interviews and observations with adults ranging from those with no memory problems to those 2 years post-diagnosis [12]. These two studies contributed to the development of the PRIDE intervention, along with existing literature, which was then tested to inform present feasibility randomised controlled trial. Findings from this feasibility RCT will be used to inform on whether, a definitive randomised trial comparing the clinical and cost-effectiveness of the self-management PRIDE intervention is indicated. 

### Aims and objectives

The aim of the PRIDE feasibility randomised controlled trial is to investigate the feasibility and acceptability of conducting a future large-scale definitive randomised controlled trial (RCT) to compare the clinical and cost effectiveness of the PRIDE intervention delivered in addition to usual care with usual care only for people with mild dementia. The objectives of the study are to:

1. Determine the feasibility of recruitment and acceptability of randomisation
2. Refine the eligibility criteria for a future definitive RCT
3. Determine the relevance and acceptability to patients / clinicians of the trial intervention
4. Determine the acceptability to patients / clinicians of the trial procedures
5. Assess the ability of NHS sites to deliver the intervention and assess training and support needs
6. Evaluate treatment fidelity when delivered through NHS services
7. Determine the services and interventions provided as usual care and evaluate methods for measuring this
8. Assess outcome completion rates and determine the relevance and acceptability of a range of clinical outcome measures and obtain data to inform selection of the primary outcome for a future RCT
9. Evaluate the utility and acceptability of resource use questionnaires for use in an economic evaluation alongside a future RCT
10. Conduct a comparative micro-costing of the PRIDE intervention and usual care
11. Estimate the sample size required for a definitive study

## Methods/Design

**Fig. 1:**
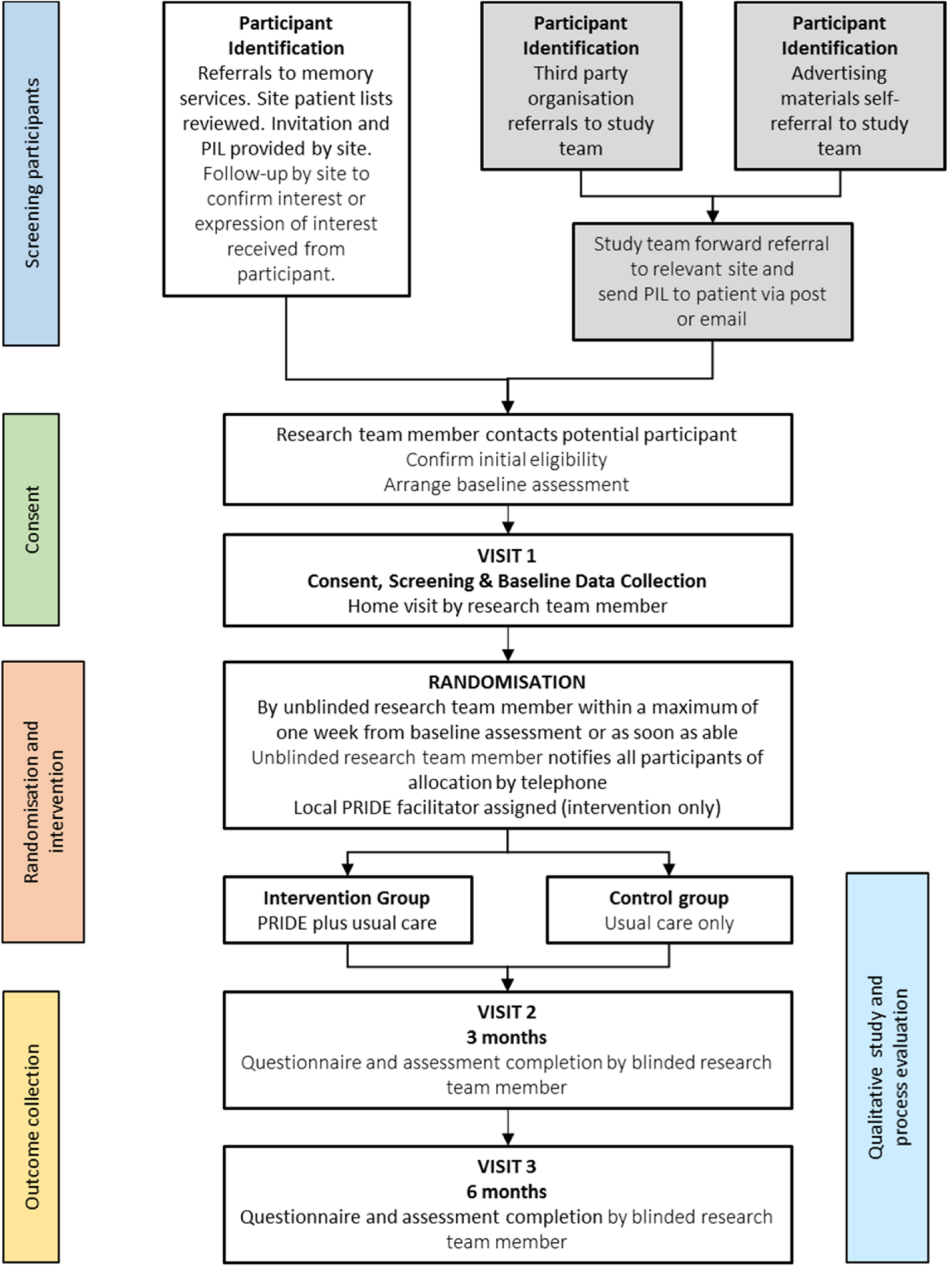
Participant pathway flowchart

The PRIDE study is a prospective, parallel, two-arm, multicentre randomised feasibility trial with participants individually allocated on a 1:1 ratio to treatment with either usual care plus the PRIDE intervention, or usual care alone. Participants will be followed up for up to 6 month’s post-randomisation. Embedded qualitative research will be used to explore the experiences of study participants, their supporters, and of facilitators delivering the interventions Fig 1.

### Participants

Recruitment is taking place in six secondary care sites in England: Humber Teaching NHS Foundation Trust, Derby Teaching Hospitals NHS Foundation Trust, North West Boroughs Healthcare NHS Foundation Trust, Oxford Health NHS Foundation Trust, Central and North West London NHS Foundation Trust and Leicestershire Partnership NHS Trust.

Participants are identified for recruitment into the trial in the following ways:

#### NHS recruitment pathway

Participants are being identified and recruited from NHS services for people with dementia within participating secondary care trusts. Potentially eligible participants are identified from patient lists of the participating services and other clinical settings and records screened by a member of the usual care team in order to ascertain initial suitability for the trial. The initial approach is being made by a member of the patient’s usual care team and is taking place either face-to-face or by invitation letter.

#### Join Dementia Research recruitment pathway

Through ‘Join Dementia Research’ (JDR), which is an online self-registration service that enables volunteers with memory problems or dementia, carers of those with memory problems or dementia and healthy volunteers to register their interest in taking part in research JDR is funded by Department of Health working in partnership with the charities Alzheimer Scotland, Alzheimer’s Research UK and Alzheimer’s Society and is Health Research Authority (HRA) endorsed.

There are two methods people with dementia can join the trial through JDR. First, potential participants may search the JDR database and can contact the coordinating centre directly once verbal consent is obtained to pass on contact details to participating sites. Second, sites are searching the JDR database and are contacting registered potential participants. Initial screening is being undertaken against non-clinical eligibility criteria and a patient information sheet about the study is being sent. Potential participants identified through JDR must be resident in a catchment area of one of the participating sites. Those who are not are advised that they do not meet the eligibility criteria for the study.

#### Self-referral recruitment pathway

Participants are also able to self-refer directly to the local research teams or the coordinating centre. Potential participants may become aware of the study through relevant local and national charities and patient organisations and through general promotion of the trial via posters.

### Eligibility

Participants are eligible for the study if they:

- are a resident within the catchment area of one of the participating NHS sites
- are aged over 18 years
- meet the Diagnostic and Statistical Manual of Mental Disorders-Fourth Edition (DSM-IV) [13] criteria for dementia of any type, including Alzheimer’s, vascular, Lewy body type and mixed
- are able to engage with and participate in the intervention in the judgement of the investigator or designee,
- are able to provide informed consent in the judgement of the investigator or designee
- are able to read and communicate in English

In addition, to be eligible for randomisation the participant must be assessed as having mild dementia, defined as a score of 0.5 or 1 on the Clinical Dementia Rating Scale [14]. The exclusion criterion is living in institutional care.

- Participants may take part with or without a supportive other. If taking part, the supporter will be eligible for the study if they are:
- aged 18 or over
- able to engage with and participate in the intervention
- able to provide informed consent and
- able to read and communicate verbally in English

### Usual care

All participants will receive the services and interventions usually available to people with dementia and their family at the participating sites. This will naturally vary between and within centres and may change over time.

### Intervention

In addition to usual care, participants allocated to the intervention will receive the PRIDE intervention, encompassing manualised social, physical and cognitive domains for people in the early stages of dementia. The manual is used to guide participants during three facilitated sessions of between 60 and 90min, held over 2 months. The trained facilitator will ensure that the intervention is tailored to meet the individual’s needs. The objectives of the PRIDE intervention are:

1. To promote independence and facilitate living well with dementia.
2. To enable the participant to maintain an active lifestyle (e.g. exercise).
3. To aid the participant to have a healthy lifestyle (e.g., smoking cessation).
4. To encourage the participant to maintain cognitive activities.
5. To provide sign posting to local services and resources about social, mental, and physical activities and healthy lifestyle opportunities.
6. To help the person maintain their social roles.

The PRIDE manual is available to participants in a paper or electronic (web-based) version. Participants may choose either the paper or electronic version, or both. The manual outlines chapters on communication, social connections, decision-making, keeping socially, mentally, and physically active, finding a balance in activities, receiving a diagnosis, and keeping healthy. Each participant is being offered three sessions with a facilitator who will tailor the intervention to their individual needs. Session 1 identifies existing lifestyle choices, activities and routines valued by the participant and identifies novel hobbies or everyday tasks they might profit from and resources to support this. The facilitator helps the participant identify social supports and explore whether the person feels they have agency in everyday decision-making, and how to create more opportunities for supported, but independent decision-making. The facilitator will introduce the person to the intervention manual and together plan a goal(s) to work on over the next two months. Session 2 reviews progress from session 1. Goals may be refined according to the participant and supporters’ experience of implementation and any needs which may have arisen in the first month. New goals may also be set. Session 3 reviews progress again and final session focusses on developing plans to maintain the changes placed during previous sessions. Sessions 2 and 3 may also involve the facilitator participating in activities out in the community with the person or participant and supporter based on their choices.

### Adherence

Fidelity checks will take place to assess how well the PRIDE intervention is being delivered according to the intervention protocol, facilitator training and manual. Checks will adhere to an intervention fidelity framework (Table 1 Change Consortium [15, 16] using the following quality assurance parameters: using the following quality assurance parameters:

**Table 1:** Fidelity assessment strategy

Goal	Description	Fidelity
Monitoring facilitator training		
Standardised training	All facilitators receive the same training programme	• Training delivered by the same trainer(s) • Attendance registers for training • Training observation checklists
Facilitator skill acquisition	All facilitators participate in the training in a similar way Did training equip facilitators with required skills	• Completion of training exercises • Training observation checklist • Focus group
Monitoring intervention delivery		
Comparable treatment	All participants receive the same programme	• Offer of 3 sessions • Attendance register for 3 sessions • All participants receive a manual
Risk to implementation	Recruitment of suitable facilitators	• Facilitator job description
Standardised delivery	All facilitators using the same techniques and content from the manual and training	• Intervention delivery checklist • Focus group
Minimise drift in skills/delivery	Adherence to training content and delivery across sites	• Focus group
Monitoring receipt of intervention		
Participant attendance and engagement	Numbers of participants attending sessions Participants identifying a goal(s)	• Attendance register for 3 sessions • Register of topics/goals covered from the manual • Focus group

#### Training

All facilitators delivering the PRIDE intervention attended a 1-day standardised training session. Delivery and receipt of training was observed and rated by two researchers (the lead for fidelity and one other member of the research team) using a bespoke training observation checklist. For the purposes of comparison, trainees were asked to rate the training according to the same criteria using a simplified checklist. The checklists listed core skills and key criteria identified out of the content of the training and the manualised intervention. Facilitator skills and understanding of the intervention was measured through training delivery techniques such as active participation as well as observed behaviours such as skill acquisition and reflection via the checklist.

Analysis of resulting data will determine inter-rater reliability between coders to establish the extent to which they attribute the same score to the same variable. Frequencies will be used to determine the extent to which the training maintained fidelity to what was intended. Similar methods have been used in previous studies [17].

#### Delivery and receipt

To ensure comparable treatment between sites and within dyads at sites, registers are being used to monitor adherence to the intervention. Quality of facilitation is managed using a generic job description.

To assess facilitator adherence to the manualised intervention and participant receipt of the intervention, focus groups will be held with a sample of facilitators and with participants and supporters respectively. Facilitators are also completing a register of attendance for each participant including number of sessions attended and topic covered during each session. This will demonstrate participant adherence to the intervention as well as adherence to the manual.

### Outcomes

#### Feasibility outcomes

The primary outcome of this study is the feasibility of conducting a full RCT of the PRIDE intervention, determined by a range of measures related to the study objectives. The feasibility outcomes, which will be measured to meet the objectives of this study, are shown in Table 2.

**Table 2:** Feasibility objectives and outcomes

Feasibility objectives	Feasibility outcomes
1. Determine the feasibility of recruitment to a large-scale RCT	a. Aggregate data on potential participants within NHS services b. Number of patients assessed for eligibility / consented / randomised c. Number and proportion of potential participants identified through NHS services, Join Dementia Research and by self-referral who are eligible d. Reasons for non-inclusion / non-eligibility e. Monthly recruitment rate per site f. Barriers and facilitators to recruitment (focus groups)
2. Refine the eligibility criteria for a future definitive RCT	a. Number of screening failures for eligibility, post-consent b. Participant and facilitator report (focus groups)
3. Determine the acceptability to patients / clinicians of randomisation	a. Proportion of eligible patients that consent to randomisation b. Reasons for non-consent c. Participant and facilitator report (focus groups)
4. Determine the relevance and acceptability to patients / clinicians of the trial intervention	a. Premature discontinuation or non-attendance of treatment and reasons b. Feedback from participants and site staff delivering the intervention c. Participant and facilitator report (focus groups)
5. Determine the acceptability to patients / clinicians of the trial procedures	a. Proportion of approached NHS sites that agree to participate in the trial and reasons for non-participation b. Proportion of eligible patients that consent to randomisation c. Reasons for non-consent d. Withdrawals and losses to follow-up and reasons e. Feedback from participants and staff (focus groups)
6. Assess the ability of NHS sites to deliver the intervention	a. Measures of the feasibility of delivering the PRIDE intervention within NHS settings: i. Number / grade / experience of staff within the service ii. Staff turnover iii. Time to treatment initiation b. Measures of the recruitment and retention of PRIDE facilitators during the study treatment period c. Barriers to treatment delivery per protocol (focus groups)
7. Assess training and support needs for NHS staff delivering the intervention	a. Feedback on training delivered (focus groups) b. Support offered / accepted (e.g. log of calls and emails to central support lines)
8. Evaluate treatment fidelity when delivered through NHS services	a. Measures of treatment fidelity including: i. Adherence to intervention manual ii. Uptake of activities b. Feedback from participants and staff (focus groups)
9. Determine the services and interventions provided as usual care and evaluate methods for measuring this	a. Post-diagnostic care pathway b. Services available c. Uptake of services
10. Assess follow-up and outcome completion rates	a. Response rate to follow-up assessment b. Questionnaire completion rates c. Amount of missing questionnaire data at item and scale levels
11. Determine the relevance and acceptability of a range of clinical outcome measures and selection of the primary outcome for the main trial	a. Completion rates and reasons for non-completion / missing data b. Estimates of clinically important differences, variance and sensitivity to change for the clinical outcome measures c. Direct questions to participants regarding relevance of measures
12. Evaluate the utility and acceptability of resource use questionnaires for use in an economic evaluation alongside a future RCT	a. Completion rate and reasons for non-completion / missing data
13. Comparative micro-costing of PRIDE intervention and usual care	a. Staff time and resources for delivery of PRIDE intervention b. Other service use
14. Estimate the sample size required for a definitive study	a. Primary outcome selection b. Variability in the outcome c. Withdrawals and losses to follow-up
15. Determine the resources required for a full trial	a. Sample size, recruitment rate (number of sites / recruitment period), staffing and resources (for recruitment, treatment and follow-up)

#### Clinical outcomes

Clinical outcomes will be measured to assess the relevance and acceptability of these outcomes for use in a future definitive RCT and to obtain information to inform selection of the primary outcome measure for a future trial. The following clinical outcomes will be assessed at baseline and at follow-up visits completed at 3 and 6 months post-randomisation. Participants randomised after 30 June 2019 will not have a 6-month follow-up visit. For these participants, the follow-up visit at 3 months will be their final visit:

#### Outcomes for the person with dementia

- Activities of Daily Living measured using the Lawton Instrumental Activities of Daily Living (IADL) Scale [18].
- Health-related quality of life measured using the EuroQoL Quality of Life Questionnaire (EQ-5D-5 L) [19]
- Quality of life measured using the DEMQOL [20]
- Mood measured using the Geriatric Depression Scale (GDS) – short form [21]
- Cognition measured using the Standardised Mini Mental State Exam (S-MMSE) [22]
- Wellbeing measured using the Control, Autonomy, Self-realisation and Pleasure (CASP) questionnaire [23]
- Quality of relationships measured using the Impact on Participation and Autonomy Questionnaire for older people (IPAQ-O) [24]
- Positive emotions measured using the Positive Psychology Outcome Measure (PPOM) [25]
- Social engagement measured using the number of social contacts and leisure activities per week
- Global change (assessed by the person with dementia and the supporter). At each follow-up time point, participants will be asked to provide a rating of their perceived change in relevant domains (wellbeing and sense of independence) since baseline, using a 5-point ordinal scale (much better, a bit better, no change, a bit worse, much worse).

#### Outcomes for supporters (if participating)

- Health-related quality of life measured using the EuroQoL Quality of Life Questionnaire (EQ-5D-5 L) [19]

#### Health economic outcomes

- Resource use measured using a modified version of the Client Service Receipt Inventory (CSRI) for dementia [26]. Participants and supporters will be asked to provide a retrospective report of resource use in the preceding 3 months.
- Quality Adjusted Life Years (QALYs) calculated using health related quality of life data collected through the EQ-5D-5 L and DEMQOL.
  Micro-costing of the PRIDE intervention determined by collection of information from participating sites regarding the staff time and resources for delivery of the PRIDE intervention, including implementation, training and delivery.

### Sample size

As this is a feasibility study, a formal sample size calculation for between group comparisons of a primary outcome is not appropriate. A sample size of 75 randomised participants will allow estimation of recruitment fraction with a margin of error (half-width of 95% confidence interval) of around 8 percentage points, and retention of 12 percentage points.

### Recruitment

Recruitment started on 22 November 2018 and will end on 30 June 2019. Participants randomised after 30 June 2019 will not have a 6-month follow-up visit. For these participants the follow-up visit at 3 months will be their final visit. Participants will be recruited from six secondary care sites in England. Whether identified in memory clinics, via PICs, through JDR or by self-referral, the investigator or their nominee, e.g. from the research team or a member of the participant’s usual care team, will inform the potential participant of all aspects pertaining to participation in the study and a written Participant Information Sheet will be provided.

Participants identified through JDR or by self-referral will be registered as outpatients of the memory clinic of the participating NHS trust at enrolment in the trial.

Participants will be advised that they can choose to take part either with a supportive other or on their own. If the participant chooses to take part with a supporter they will be asked to nominate a person, who if eligible, will be invited to join the study (note that it will also be possible for a supporter to assist the participant, without participating in the study in their own right). The potential supporter will be provided with written information about the study and be asked to attend the screening and baseline visit with the participant, where informed written consent will be obtained.

### Consent

Trial participants have mild dementia, and therefore are generally expected to be competent to give informed consent for participation, provided that appropriate care is taken in explaining the research and sufficient time is allowed for them to reach a decision. If it is helpful for a supporter to be involved, we would aim to ensure that this is done wherever possible. In seeking consent, we will follow current guidance from the British Psychological Society on evaluation of capacity [27]. In this context, consent has to be regarded as a continuing process and will be continually checked through discussion with participants during the assessments. If the participant’s level of impairment increases during the course of their involvement in the study to the extent that, in the judgement of the investigator (or designee), they do not have capacity to provide continued informed consent for their ongoing participation in the trial at that time, the research activity would be discontinued.

Where a supporter is also participating in the trial, the supporter will be asked to provide written informed consent for their own participation in the trial. The supporter’s decision to participate or not will not affect involvement of the participant. Supporters may decline consent to participate in the trial on their own behalf but be present during and intervention sessions and researcher follow up appointments in order to support the person with dementia, should they request this. In this case, their involvement will be recorded but no further details or data will be collected.

### Follow-up

Each participant will be in the trial for a maximum of 6 months, from randomisation to final follow-up. Table 3 describes the study procedures and assessments at each time point including screening and baseline. All baseline and follow-up visits will be completed face-to-face with a researcher.

**Table 3:** Study procedures and assessments

Months	0		0–2	3	6^f^
Study Procedures and Assessments: Participants	Visit 1	RANDOMISATION	Intervention period	Visit 2	Visit 3
	Baseline			Follow-up	
Initial eligibility screen^a^	X		*Intervention:* PRIDE intervention (3 sessions with a PRIDE facilitator) in addition to usual care *Control:* Usual care only		
Informed consent^b^	X				
Demographic information	X				
Post-consent eligibility screen: Clinical Dementia Rating (CDR) Scale^c^	X				
Lawton IADL Scale	X			X	X
EuroQoL Quality of Life (EQ-5D-5 L)	X			X	X
DEMQOL	X			X	X
Geriatric Depression Scale (GDS)	X			X	X
Standardised Mini Mental State Exam (SMMSE)	X			X	X
Control, Autonomy, Self-realisation and Pleasure (CASP-19)	X			X	X
Impact on Participation and Autonomy (IPAQ-O) – Social Relations Sub-Scale	X			X	X
Positive Psychology Outcome Measure (PPOM)	X			X	X
Social Engagement Checklist	X			X	X
Global Change Measure				X	X
Client Service Receipt Inventory (CSRI)	X			X	X
Focus groups with facilitators and participants / supporters				X^d^	
Study Procedures and Assessments: Supporters	Visit 1			Visit 2	Visit 3^f^
	Baseline			Follow-up	
Informed consent	X				
Supporter questionnaire: EuroQoL Quality of Life (EQ-5D-5 L)^e^	X			X	X
Global Change Measure^e^				X	X
Supporter questionnaire: Client Service Receipt Inventory (CSRI)^e^	X			X	X

^a^Participants not included in the trial will be recorded on the screening log, with reasons for non-inclusion documented

^b^All participants providing consent will be enrolled on the trial database, with demographic details and screening assessments recorded

^c^Participants who are identified as ineligible for randomisation during the post-consent eligibility screen will be defined as screen failures and will not proceed to randomisation

^d^Separate focus groups will be completed with a facilitator and a subset of participants and supporters

^e^Supporter questionnaires will be passed to supporters during participant visits (if present) for completion during the visit or will be posted with a prepaid envelope for return to the Coordinating Centre

^f^Participants randomised after 30th June 2019 will not have a 6-month follow-up visit, and their last visit will be at visit 2

The duration of the trial is expected to be up to 24 months from recruitment of the first participant to reporting.

### Withdrawal

Participants can withdraw from the trial at any time without affecting their current or future care. Reasons for withdrawal will be sought and recorded.

Participants in the intervention group may discontinue the PRIDE intervention at any time. Reasons for discontinuation from the intervention will be recorded. Participants who prematurely discontinue the trial intervention will be asked to remain in the trial for follow-up. If participants who prematurely discontinue the trial intervention choose to also withdraw from trial follow-up, the reason for withdrawal will be requested, and documented where given.

Supporters may also withdraw from the trial as a whole or choose to discontinue their involvement in the trial intervention. Doing so will not affect the participants continuation in the trial. The withdrawal or discontinuation of supporters will be recorded, with reasons for withdrawal or discontinuation requested, and documented where given. If the participant wishes to identify an alternative supporter to participate in the trial with them, they will be able to do so. The alternative supporter will provide consent to participate and the change of supporter will be documented.

### Randomisation

Participants will be allocated at the individual level to intervention or control on a 1:1 ratio using minimisation with a probabilistic element. The minimisation variables will be study site, sex, age (< 80 or ≥ 80) and medication for dementia (any versus none). The allocation algorithm will be created by the Nottingham Clinical Trials Unit (NCTU) in accordance with their Standard Operating Procedure (SOP) and held on a secure server. The investigator or authorised designee will use the remote, internet-based randomisation system to obtain the treatment allocation for each participant.

Following randomisation, participants will be notified of their treatment allocation by an unblinded member of the research team and their GP will be notified of their involvement in the trial.

### Blinding

Due to the nature of the intervention, blinding of treatment allocation is impossible for participants and the staff delivering it. The study outcome data at all the time points will be collected by members of the local research team who will be blind to group allocation. The researchers conducting the outcome assessment visits will record instances of unblinding. The trial statisticians will remain blinded to treatment allocation until after database lock.

### Adverse events

No adverse reactions have been identified in previous trials of social and psychological interventions for people with dementia [9, 28, 29]. The risks of the current trial have therefore been assessed as low and there are unlikely to be adverse events resulting from the trial. For this reason, adverse events (AEs) and serious adverse events (SAEs) will not be routinely collected for this trial.

The assessments for participants will be limited in duration and not unduly long or stressful. Researchers and facilitators delivering the intervention will be trained to identify and deal with any distress exhibited by trial participants during trial activities and will make referrals (e.g. to the participant’s GP) if needed.

### Data collection, management and analysis

Data collection and clinical assessments will be in person with the researcher and conducted in the participant’s home (or other suitable community or NHS venue, as appropriate, depending on participant preference). Participant questionnaires at baseline, follow-up at 3 and 6 months will be self-completed by participants during the visits. If the participant chooses to take part with a supporter, the designated supporter will be asked to complete questionnaires also at baseline and follow-up at 3 and 6 months. If the supporter is not present during the follow-up visit, the questionnaire may be posted to them.

Questionnaire data will be entered onto the trial database which includes programmed validation checks. Checks will include missing data (including missing forms), out of range values, illogical entries and invalid responses. Data collection and retention rates will be monitored by the Trial Management Group (TMG) throughout the trial.

### Statistical analyses

Data analysis will primarily be descriptive to address the feasibility aims of the study. All analyses will be documented in a Statistical Analysis Plan, which will be finalised prior to database lock. Feasibility outcomes will be estimated using descriptive statistics (with 95% confidence intervals [CI] if relevant) and will include recruitment rates, follow-up rates, amount of missing data, and intervention adherence. The rate of protocol adherence will be reported within the intervention group in terms of participants who adhere to the intervention they were allocated to receive and who comply with the scheduled treatment visits.

Key baseline characteristics (age, gender) will be compared between trial participants and the ineligible and non-consenting patients, to ascertain adequacy of inclusion/exclusion criteria and likely generalisability of the trial to the required targeted population.

Similarly, we will compare the key patient characteristics between those followed-up and those lost to follow-up and investigate how similar this is across the treatment arms to assess possible attrition bias in data collection.

A baseline table will compare important demographic and clinical characteristics between the two treatment arms. It is not an objective of the feasibility study to obtain estimates of intervention effect on clinical outcomes and so the clinical outcomes will be presented descriptively.

Global change outcomes will be used to categorise improvers / non-improvers for anchor-based analysis of minimum clinically important differences and responsiveness to change of relevant, related outcome measures.

## Trial management and oversight

Nottingham Clinical Trials Unit (NCTU) is the coordinating centre and responsible for all trial management activities. Trial oversight will be provided by an independent Trial Steering Committee (TSC) who will monitor trial progress and assess feasibility. As the trial interventions and procedures have been assessed as low risk, safety oversight will be performed by the TSC without the need for a separate independent Data Monitoring Committee.

The Trial Management Group (TMG) responsible for the day-to-day delivery of the trial will also meet monthly and report to the TSC on progress.

## Qualitative sub-study

Facilitator and participant (supporters, where present) focus groups will be arranged to explore perspectives on the intervention and investigate a range of themes including, but not limited to:

- Acceptability of the intervention (manual, sessions, facilitation)
- Barriers and facilitators to delivery of the intervention
- Experience of the intervention
- Factors that may mediate or moderate the effectiveness of the intervention
- Skills and competencies required to deliver the intervention
- Barriers and facilitators to continued use of the intervention
- Acceptability of the outcome measures (quantity, content, ability to complete)
- Acceptability of the trial procedures (recruitment, consent, randomisation and activities throughout the follow-up period)

Utilising a focus group approach will help people to identify and clarify their views in relation to others who have experienced the same intervention [30] and support sharing of their ideas and similar or different opinions.

A schedule of topics and questions will help guide discussion to produce final themes [31]. Field notes will be used to record discussions and agreement. Results will be used to explore potential explanations for the quantitative findings and identify emergent factors that influence the uptake and impact of the intervention and other trial procedures for a future large trial.

## Protocol amendments

All methods described here reflect the current study protocol (V2.1 dated 19June2019). This protocol Additional file 1 conforms to the SPIRIT recommendations [32]. See Table 4 for a summary of protocol amendments. All amendments to the protocol have been approved by the trial sponsor, Research Ethics Committee and local R&D departments prior to implementation.

**Table 4:** Summary of amendments to the PRIDE feasibility study protocol

Protocol	Date	Summary of changes
2.0	20 Dec 2019	• GP practices added as Participant Identification Centres • GHQ-12 questionnaire removed to reduce the burden on participant and supporters in completing questionnaires • Allowed sites the flexibility to manage the Join Dementia Research database to identify potential participants
2.1	22 May 2019	• Extension of the recruitment period for a month if required • Participants randomised after 30th June 2019 will only have a 3-month follow-up visit

## Confidentiality

Participant confidentiality will be ensured by allocating participants a unique identification number to correspond to treatment data in the computer files.

If information is disclosed during the study that could pose a risk of harm to the participant or others, the researcher will discuss this with the CI and where appropriate report accordingly.

Data generated as a result of this trial will be available for inspection on request by appropriate organisations and bodies such as the REC and the regulatory authorities.

## Discussion

With the prevalence of dementia growing as the population ages, the imperative to develop novel interventions which are acceptable and effective is globally recognised by the governments and health departments of nations worldwide (e.g. UK Department of Health, 2016; US Department of Health and Human Services, 2012). Increased public awareness and a drive toward early diagnosis can lead to dementia being identified while still mild, which presents the opportunity to offer interventions the person with dementia (and their family) can actively play a part in. The development of psychological interventions promoting social inclusion, dignity and the positive contributions to society will reduce the current challenges that are faced by people with mild dementia. Many people with dementia remain undiagnosed and memory services see many people in the early stages of dementia who are independent and are able to participate in community activities. Therefore, there is a need to improve early diagnosis, reduce discrimination and develop an intervention with improved access to information and advice.

The provision of psychosocial interventions built on self-management in this early stage may lead to individuals being able to maintain some independence and remain a part of their community [33]. Indeed, people with dementia have the agency and desire to shape their lives [34] and this can be capitalised on. This study may also give a potential solution and share post-diagnostic experiences of many people with dementia and carers [35]. As part of the research programme social and lifestyle changes were investigated to reduce risk and by understanding the social impact of dementia and an effective PRIDE intervention was developed. The intervention manual focuses on promoting independence and quality of life for people with mild dementia family and friends that support them.

Through the PRIDE programme we have created an intervention we hope will be an effective tool to help support independence for longer to reduce stigma and social exclusion, and to improve communication and wellbeing. The robust development and evaluation of such an intervention is necessary [36].

A comparison of the PRIDE social intervention plus usual care with usual care alone has been identified as an important research question. However, there is uncertainty if the NHS services are able to deliver the intervention and are willing to recruit, and whether patients will be willing to be randomised to a study with several intervention sessions. The cost implications will be another uncertainty alongside any social circumstances of the patient and carers.

The PRIDE feasibility study will provide data essential to design and conduct a larger trial comparing the outcomes and costs of the PRIDE intervention with usual care to promote and enhance independence and quality of life for people with mild dementia and carers that support them.

## Trial status

Protocol version 2.1 22 May 2019. Recruitment commenced in November 2018 and is expected to continue until the end of June 2019.

## Supplementary information

**Additional file 1.** VITA SPIRIT 2013 Checklist: recommended items to address in a clinical trial protocol and related documents.

## Data Availability

The datasets generated during and/or analysed during the current study will be available upon request from Nottingham Clinical Trials Unit. Anonymised, participant-level data will be available following publication of the results by the trial team.

## Abbreviations

AE: Adverse event
CI: Chief investigator
GP: General practitioner
HRA: Health Research Authority
JDR: Join Dementia Research
NCTU: Nottingham Clinical Trials Unit
NDS: National Dementia Strategy
NHS: National Health Service
PRIDE: Promoting Independence in Dementia
R&D: Research and development department
RCT: Randomised controlled trial
REC: Research ethics committee
SAE: Serious adverse event
SOP: Standard operating procedure
TMG: Trial management group
TSC: Trial steering committee
